# Attacking Cancer Progression and Metastasis

**DOI:** 10.3390/ijms24097858

**Published:** 2023-04-26

**Authors:** Ľuba Hunáková

**Affiliations:** Institute of Immunology, Faculty of Medicine, Comenius University in Bratislava, 813 72 Bratislava, Slovakia; luba.hunakova@fmed.uniba.sk

This Special Issue, focused on a collection of papers on “attacking cancer progression and metastasis”, is devoted to communicating current knowledge about the cellular and molecular mechanisms involved in cancer progression and metastasis, as well as suggesting new targets for possible future therapeutic interventions. It aims to provide ample scope for new ideas on how to block or weaken processes of cancer progression to its final stage. Nine interesting scientific papers from basic research covering a broad spectrum of cancer types (from breast and cervical through colorectal to gastrointestinal, thyroid, and pancreatic cancer) and seven reviews offer an extensive view on various aspects of possible targetable mechanisms leading to possible future interventions.

In the research article “Anti-Stem Cell Property of Pterostilbene in Gastrointestinal Cancer Cells”, Shiori Mori et al. [1] showed that Pterostilbene (PTE), a natural sterbenoid contained in blueberries, causes dose-dependent inhibition of cell proliferation, sphere-forming ability, and stem cell marker expression in three gastrointestinal cancer cell lines (CT26, HT29, and MKN74). These antitumor effects of PTE can be considered useful in cancer treatment.

Monika Barathova et al. [2] described a crosstalk between beta-blockade by propranolol (a non-specific blocker of beta-adrenergic receptors) and the tumour microenvironment. Propranolol reduced the metastatic potential, viability, and proliferation of colorectal cancer cells cultivated in multicellular spheroids. Furthermore, it decreased the ability of tumour cells to adapt to hypoxia by reducing the levels of HIF1α and carbonic anhydrase IX. Chun-Min Su et al. [3] reported an association between the magnolol-inhibited progression of colorectal cancer in vitro and in vivo and the induction of apoptosis through extrinsic/intrinsic pathways and the blockage of PKCδ/NF-κB signaling.

Amy Scholze et al. [4] proved that the oncogene MYC target v1 and v2 scores were associated with tumor aggressiveness and poor prognosis in ER-positive primary tumors, as well as in metastatic breast cancer. In cervical cancer, the HPV16 E6 oncoprotein, a member of the human papillomavirus (HPV) family, facilitated tumour growth and angiogenesis, as evidenced by Seung Bae Rho et al. [5]. Interferon regulatory factor-1 (IRF-1) suppressed HPV16 E6-induced tumourigenesis and angiogenesis.

The up-regulation of the receptor for advanced glycation end products (RAGE), in the absence of stimulation by external ligands, has been shown by Priyanka Swami et al. [6] to modulate cell proliferation and migration differently in pancreatic cancer cells, and to partly regulate epithelial-to-mesenchymal transition (EMT).

Margarite Knyazeva et al. [7] used coupled experimental and computational approaches to identify reciprocal dysregulation of miR-146b and miR-451 as important attributes of follicular cell malignant transformation and follicular thyroid cancer progression. They suggested that the evaluation of the combined dysregulation of miRNAs relevant to invasion and metastasis can help to distinguish truly malignant follicular thyroid cancer from indolent follicular adenoma.

Targeting collagen prolyl 4-hydroxylase 1 (C-P4H1) is considered a potential therapeutic strategy for collagen-related cancer progression and metastasis. Shike Wang et al. [8] developed a high-throughput screening assay for C-P4H1 inhibitor screening by quantifying succinate, a byproduct of C-P4H-catalyzed hydroxylation. They performed high-throughput screening with the FDA-approved drug library and identified several new C-P4H1 inhibitors, including Silodosin and Ticlopidine.

The role of methionine aminopeptidase 2 (MetAp2), an intracellular enzyme known to modulate angiogenesis, in lymphangiogenesis has been described by Rawnaque Esa et al. [9]. The genetic and biochemical manipulation of MetAp2 confirmed the dual activity of the enzyme in both vascular and lymphatic remodulation in cell function assays and in a zebrafish model. The authors suggested that MetAp2 inhibitors can be effectively used as anti-metastatic broad-spectrum drugs.

Among the reviews published in this Special Issue, Olamide T. Olaoba et al. [10] offered a review of the current role of RAGE in melanoma and concluded that targeting RAGE in melanoma could be an approach to improve the outcomes of melanoma patients.

Eleonora A. Braga et al. [11] analyzed regulatory long non-coding RNAs (lncRNAs) competing with protein-coding mRNAs for binding to miRNAs according to the model of competitive endogenous RNA (ceRNA) in ovarian cancer (OvCa). They showed that most lncRNAs acting as ceRNAs participate in OvCa progression: migration, invasion, epithelial–mesenchymal transition (EMT), and metastasis.

Sylwia Tabor et al. [12] discussed current, newly developed, and recently discovered methods that may become useful in assessing the probability of relapse in luminal breast cancer. Despite the relatively good prognosis of this predominant type of breast cancer, its heterogeneity creates problems with properly stratifying patients and correctly identifying the group at high risk of metastatic relapse.

Boris Mravec et al. [13] provided a new complex view of the role of β-adrenergic receptor signaling within the tumor micro- and macroenvironments, as well as in mediating the effects of psychosocial and spiritual environments. Furthermore, they described potential preventive and therapeutic implications.

The review “New Insights into Therapy-Induced Progression of Cancer” written by Polina V. Shnaider et al. [14] summarizes mechanisms of acquired resistance, such as secondary genetic alterations and impaired function of drug transporters and autophagy. The authors also describe less obvious molecular aspects of therapy resistance in cancers, including epithelial-to-mesenchymal transition, cell cycle alterations, and the role of intercellular communication.

Marisol Miranda-Galvis and Yong Teng [15] aimed to understand the biological processes, key events, and consequences related to hypoxia-driven metabolic adaptation of tumor cells. They also evaluated the potential therapeutic impact of hypoxia and discussed possible therapeutic strategies targeting hypoxia that would advance the current understanding of hypoxia-associated tumor propagation and malignant progression and improve the management of tumor hypoxia.

Finally, Sona Ciernikova et al. [16] summarized the current research findings on the deregulation of epigenetic mechanisms in pancreatic ductal adenocarcinoma (PDAC) and the influence of the epigenome on the dynamics of gene expression changes underlying EMT, which is responsible for the invasive phenotype of cancer cells and, therefore, their metastatic potential. They also provided an overview of the studies that uncover potentially actionable pathways.
